# Generation, analysis, and transformation of macro-chloroplast Potato (*Solanum tuberosum)* lines for chloroplast biotechnology

**DOI:** 10.1038/s41598-020-78237-x

**Published:** 2020-12-03

**Authors:** Alessandro Occhialini, Alexander C. Pfotenhauer, Taylor P. Frazier, Li Li, Stacee A. Harbison, Andrew J. Lail, Zachary Mebane, Agnieszka A. Piatek, Stephen B. Rigoulot, Henry Daniell, C. Neal Stewart, Scott C. Lenaghan

**Affiliations:** 1 Department of Food Science, University of Tennessee, 102 Food Safety and Processing Building, 2600 River Dr., Knoxville, TN 37996 USA; 2 Center for Agricultural Synthetic Biology (CASB), University of Tennessee Institute of Agriculture, Knoxville, TN 37996 USA; 3 Department of Plant Sciences, University of Tennessee, Knoxville, TN 37996 USA; 4 Elo Life Systems, Durham, NC 27709 USA; 5 Department of Basic and Translational Sciences, School of Dental Medicine, University of Pennsylvania, Philadelphia, PA USA

**Keywords:** Plant biotechnology, Biotechnology, Plant sciences, Plant biotechnology, Plant molecular biology, Plant physiology

## Abstract

Chloroplast biotechnology is a route for novel crop metabolic engineering. The potential bio-confinement of transgenes, the high protein expression and the possibility to organize genes into operons represent considerable advantages that make chloroplasts valuable targets in agricultural biotechnology. In the last 3 decades, chloroplast genomes from a few economically important crops have been successfully transformed. The main bottlenecks that prevent efficient transformation in a greater number of crops include the dearth of proven selectable marker gene-selection combinations and tissue culture methods for efficient regeneration of transplastomic plants. The prospects of increasing organelle size are attractive from several perspectives, including an increase in the surface area of potential targets. As a proof-of-concept, we generated *Solanum tuberosum* (potato) macro-chloroplast lines overexpressing the tubulin-like GTPase protein gene *FtsZ1* from *Arabidopsis thaliana*. Macro-chloroplast lines exhibited delayed growth at anthesis; however, at the time of harvest there was no significant difference in height between macro-chloroplast and wild-type lines. Macro-chloroplasts were successfully transformed by biolistic DNA-delivery and efficiently regenerated into homoplasmic transplastomic lines. We also demonstrated that macro-chloroplasts accumulate the same amount of heterologous protein than wild-type organelles, confirming efficient usage in plastid engineering. Advantages and limitations of using enlarge compartments in chloroplast biotechnology are discussed.

## Introduction

Chloroplast biotechnology has long been recognized as a relatively untapped resource for enabling metabolic engineering and crop improvement^1–3^. While metabolic engineering in plastids has been used for bioproduction of carotenoids^4^, terpenoids^5^, vaccines^6,7^, pharmaceutical drugs^8,9^ and valuable enzymes of industrial interest^10,11^, no commercial transplastomic crops have been introduced in the market. Even fewer studies have focused on plastid engineering for crop improvement, such as increasing photosynthetic efficiency^12,13^ or mitigating biotic and abiotic stresses^14,15^. Given the inherent bioconfinement of transgenes in plastids^16,17^, the lack of transgene position effects^1,18^, absence of gene silencing mechanisms, extremely high heterologous protein production^19,20^, and ability to coordinate gene expression in operons^21,22^, plastid engineering appears to be an attractive strategy. Nonetheless, there are some technological barriers that are impediments in need of addressing in order to widely implement chloroplast transformation for crop agriculture.

Together with the model species tobacco and more recently, Arabidopsis^23^, chloroplasts of few agronomically important dicotyledon crops including tomato, potato and lettuce^22,24–26^ have been successfully transformed and regenerated to homoplasmic transplastomic lines. All monocotyledon cereals have proven recalcitrant to current methods to generate stable transplastomic plants. Only few examples of transient chloroplast transformation in rice (*Oryza sativa*) have been published^27,28^. Successful chloroplast transformation in key crops, including cereals, will depend on the ability to find more efficient selection (along with selectable marker genes) and tissue culture methods to efficiently regenerate transplastomic plants.

The ability to significantly increase plastid size provides an intriguing avenue with regards to plastid biotechnology. While the small size of plastids has been shown to not be a limiting factor for biolistic transformation, assuming a strong selection system^29^, for alternative methods of plastid transformation (e.g. microinjection), an increase in plastid size would be beneficial if not necessary. For example, the installation of large DNA molecules (synthetic plastid genomes) will likely require alternative transformation strategies.

In higher plants, chloroplast division is regulated by two FtsZ protein homologous (FtsZ1 and 2)^30,31^. FtsZ1 is a tubulin-like GTPase that, in association with FtsZ2 and other division-related components, regulate chloroplast division by participating in the formation of a z-ring at the mid-organelle^32–35^. Considering that the stoichiometry between FtsZ proteins is important for their correct function in z-ring formation, the most common strategy to increase chloroplast size is through altering FtsZ1 gene expression^30,32–34^. Overexpression of *FtsZ1* in Arabidopsis was altered chloroplast division resulting in a reduction of organelle number and drastic increase in size, leading to the term “macro-chloroplast”^36^. Surprisingly, despite this extreme chloroplast phenotype, macro-chloroplast lines were indistinguishable from wild-type lines with regards to plant growth and morphology^30^. However, in a follow-up study in *Nicotiana tabacum* (tobacco) a significant growth reduction was observed under high- and low-light irradiation in lines with a severe macro-chloroplast phenotype (defined as 1–3 large macro-chloroplasts per cell)^37^. In medium-light irradiation, the growth of all macro-chloroplast lines were indistinguishable from controls^37^. In potato, macro-chloroplast lines produced alterations in both chloroplasts and amyloplasts^38^. Macro-chloroplast potato demonstrated morphological changes in starch granule size as well as increased phosphorous content in isolated starch grana compared to wild-type lines^38^. The ability to alter the size and content of non-green plastids is particularly relevant to plastid biotechnology for food crop improvement, where non-green plastids represent a primary source of nutrition (e.g. chromoplasts of tomato)^39,40^.

While methods to generate macro-chloroplasts have been established for decades, there are few examples of chloroplast engineering using these altered plastids. The first successful transformation of macro-chloroplasts in tobacco, using biolistics, demonstrated a 40% increase in chloroplast transformation efficiency relative to wild-type controls^41^. In addition, researchers demonstrated that homoplasmic macro-chloroplast plants could be regenerated in tissue culture where they accumulated a similar amount of foreign protein compared to traditional chloroplast engineering^42^. Based on this foundational work, we sought to develop macro-chloroplast potato lines specifically for the purposes of evaluating their potential for chloroplast biotechnology. We hypothesized that the use of macro-chloroplast containing leaves would increase the transformation efficiency of potato, providing a superior method for potato chloroplast engineering. Secondarily, we hypothesized that the use of macro-chloroplasts may increase the capacity for protein overproduction without negatively affecting chloroplast function. This would be particularly useful for production of plant bioreactors for high production of foreign proteins.

Specifically, in this work we engineered *AtFtsZ1* overexpressing potato lines and selected for macro-chloroplast phenotypes for use in chloroplast biotechnology. The phenotypes of macro-chloroplast lines were characterized, as well as, the efficiency of chloroplast transformation, selection, and regeneration in selected lines.

## Results

### Generation of AtFtsZ1 transgenic potato lines with enlarged chloroplast morphology

**Figure 1:**
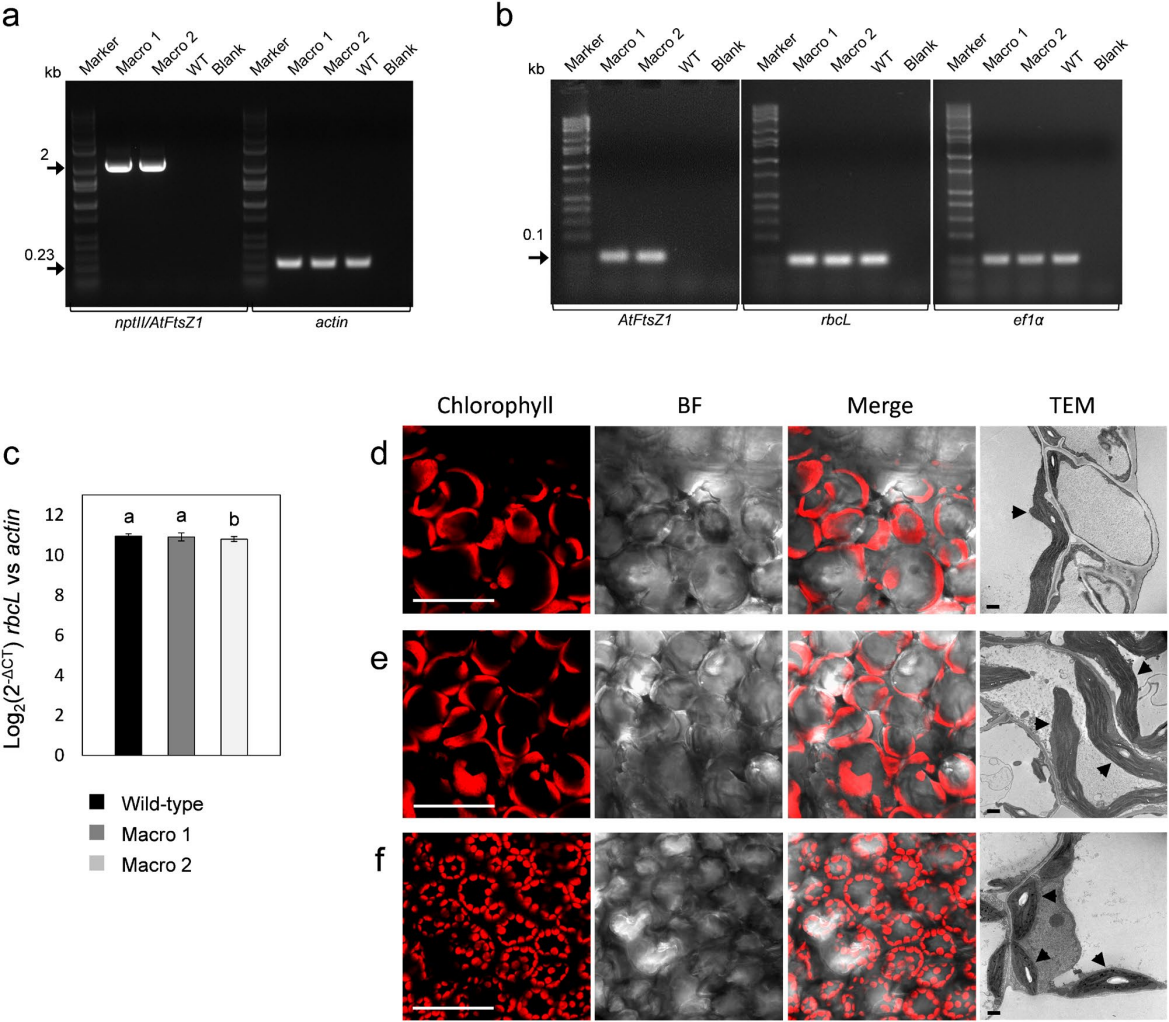
Genetic and microscopic characterization of *AtFtsZ1* over-expressing potato lines. (**a**) PCRs using a pair of primers specific for the cassette *nptII/AtFtsZ1* confirmed the presence of transgenes integrated (2 kb DNA bands) in the genome of *AtFtsZ1*-line 1 (Macro 1) and 2 (Macro 2). Primers specific for the *actin* gene were used as loading controls (0.23 kb DNA bands). (**b**) PCR reactions using cDNA preparations of macro lines (Macro 1 and 2) and primers specific for *AtFtsZ1* confirmed transgene expression in both transgenic lines (0.1 kb DNA bands). Primers for *rbcL* and *ef1α* were used as loading controls (0.1 kb DNA bands) for the plastome and nuclear genome, respectively. Wild-type samples (WT), blanks and molecular-weight markers are also shown in the gels. (**c**) Graph summarizing the Log_2_(2^−ΔCT^) values of *rbcL* (plastome) vs *actin* (nuclear genome) in total DNA preparations of macro lines (Macro 1 and 2) and wild-type controls obtained by Real-Time PCR. Results are expressed as mean ± standard deviation (SD) of 3 biological and 8 technical replicates per each biological replicate. Means were compared using ANOVA and when significant, mean separations were analyzed using Tukey HSD (p < 0.05). Statistical significance is indicated by letters (**a**,**b**). (**d**–**f**) Chloroplast morphology in *AtFtsZ1* lines and wild-type plants (WT). Confocal stack images (chlorophyll, bright field (BF), and merged images) of leaf mesophyll cells showing large chloroplasts in *AtFtsZ1* over-expressing lines (**d**,**e**, Macro line 1 and line 2, respectively) and normal chloroplast morphology in the WT control (**f**) are shown. Electron micrographs of ultrathin sections showing mesophyll cells from leaf tissue prepared by chemical fixation are also shown (**d**–**f**). Ultra-structures of macro-chloroplast morphology in *AtFtsZ1*-line 1 and 2 (**d**,**e**) along with organelles with normal morphology in wild type plants (**f**) are indicated with black arrows. Scale bars: 50 µm (confocal images **d**–**f**); 1 µm (TEM images **d**–**f**).

Nuclear genome-transformed potato lines overexpressing *AtFtsZ1* under the control of the CaMV 35S promoter were generated by *Agrobacterium*-mediated transformation. In a first screen, six independent lines were chosen for in-depth analysis from > 200 putative transgenic lines using microscopic analysis of chloroplast morphology in leaf mesophyll-derived protoplasts (Supplementary Fig. S1). Thereafter, we focused on two independent lines that had large chloroplasts (*AtFtsZ1* lines 1 and 2, Fig. S1), which appeared to be vigorous in tissue culture. These two lines were PCR-positive for the transgene (Fig. 1). The presence of the expression cassette integrated into total genomic DNA preparations was verified by amplifying the upstream *nptII* selection gene along with the downstream *AtFtsZ1* transgene (~ 2 kb DNA bands; Fig. 1a). No DNA bands were detected in wild-type samples. In both wild-type and macro-chloroplast lines, DNA bands at similar intensity were obtained for the endogenous loading control *actin* indicating unaltered amount of genomic DNA in all samples (~ 0.25 kb DNA bands; Fig. 1a). RT-PCRs using primers specific for *AtFtsZ1* confirmed transgene overexpression in the two macro-chloroplast lines (Fig. 1b). The DNA gel was normalized using RT-PCR reactions for plastome and nuclear internal controls, *rbcL* and *ef1α*, respectively (Fig. 1b). qPCR performed on total genomic DNA isolated from leaves, indicated no statistical difference in the copy number of the chloroplast genome between macro line 1 and the wild-type control (p > 0.05; Fig. 1c). On the contrary, a slight reduction was observed in line 2 (p < 0.05; Fig. 1c).

Top leaves of the canopy from 3-week-old in vitro grown macro-chloroplast lines 1, 2 and wild-type controls were observed using confocal microscopy. In leaf mesophyll cells of transgenic lines 1 and 2, significantly larger and elongated chloroplasts were observed relative to wild-type lines (Fig. 1d–f, respectively). The same lines were used for ultrastructural characterization of chloroplast morphology by transmission electron microscopy (TEM). Micrographs of ultrathin sections from mesophyll leaf cells confirmed a larger and elongated chloroplast morphology in *AtFtsZ1* transgenic potato lines (TEM; Fig. 1d,e) compared to wild-type control plants (TEM; Fig. 1f). Internal chloroplast structures, including thylakoid membranes, appeared to be morphologically normal without any apparent structural alterations (Supplementary Fig. S2).

**Figure 2:**
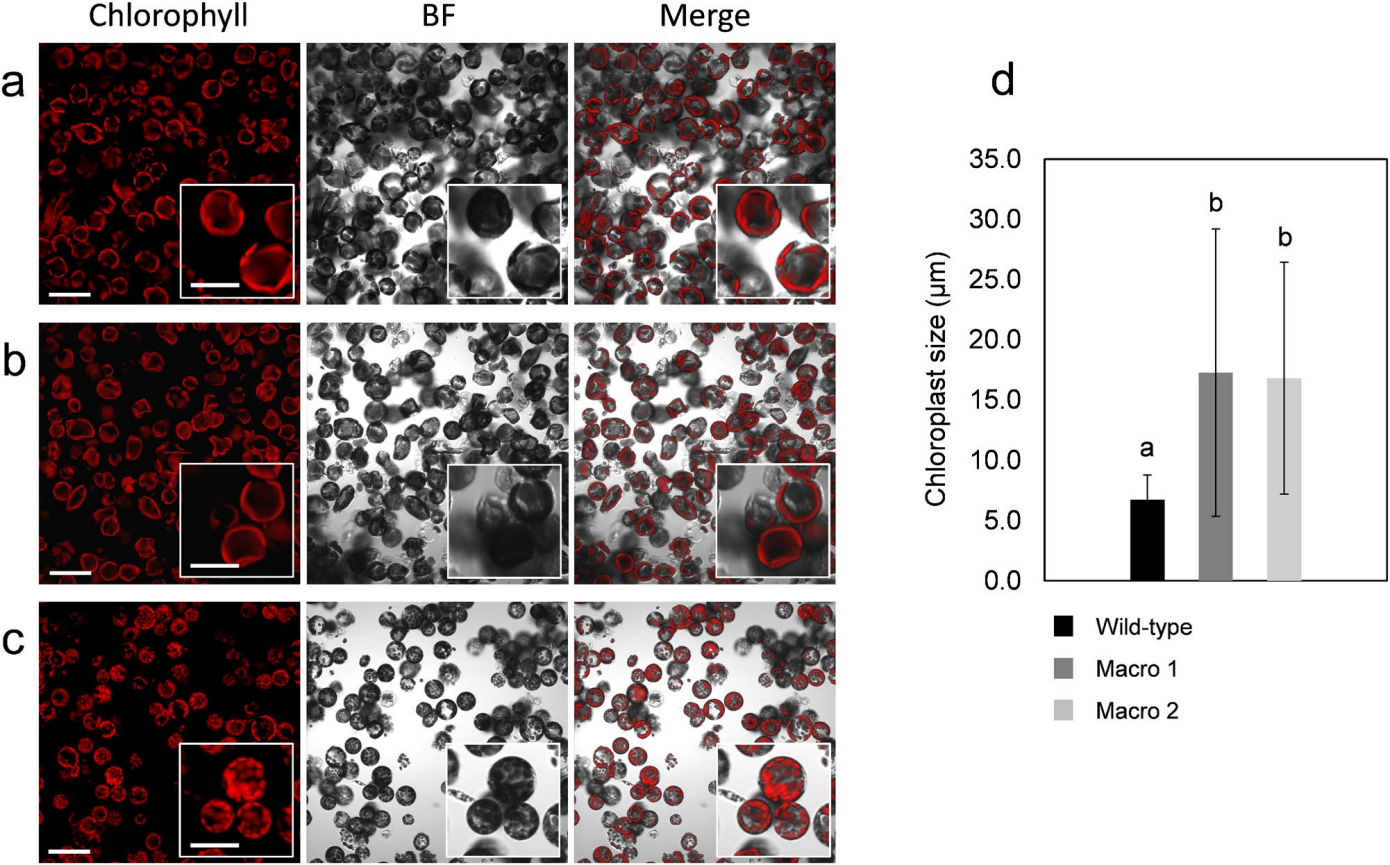
Chloroplasts size in *AtFtsZ1* over-expressing lines and wild-type controls. Confocal images of protoplasts from leaf mesophyll cells showing enlarged chloroplast phenotype in *AtFtsZ1* over-expressing lines (**a**,**b**, Macro line 1 and line 2, respectively) and normal chloroplast morphology in the wild-type control (**c**) are shown. Chlorophyll, bright field (BF), and merged images are shown. Scale bars: 50 µm (**a**–**c**); 25 µm (insert **a**–**c**). Graph representing the chloroplasts size (µm) in macro-chloroplast lines (Macro 1 and 2) and wild-type potato (d). Confocal images of isolated leaf protoplasts from transgenic lines (Macro 1 and 2) and wild-type plants have been evaluated. The results are expressed as mean ± standard deviation (SD) of 1382, 720 and 786 number (n) of chloroplasts analyzed in wild-type, Macro 1 and 2, respectively. Means were compared using ANOVA and when significant, mean separations were analyzed using Tukey HSD (p < 0.05). Statistical significance is indicated by letters (**a**,**b**).

The enlarged chloroplast phenotypes in *AtFtsZ1* transgenic lines were confirmed by measurement of organelle size from single plane confocal images from isolated protoplasts (Fig. 2a–c). Compared to chloroplast size in wild-type potato leaves (6.7 ± 2.1 µm), the organelle size in macro-chloroplast lines 1 and 2 were substantially enlarged (17.3 ± 11.9 µm and 16.8 ± 9.6 µm, respectively) (p < 0.05; Fig. 2d). The chloroplast size in *AtFtsZ1* transgenic lines 1 and 2 were not statistically different (p > 0.05; Fig. 2d).

### Phenotypic analysis of macro-chloroplast potato

**Figure 3:**
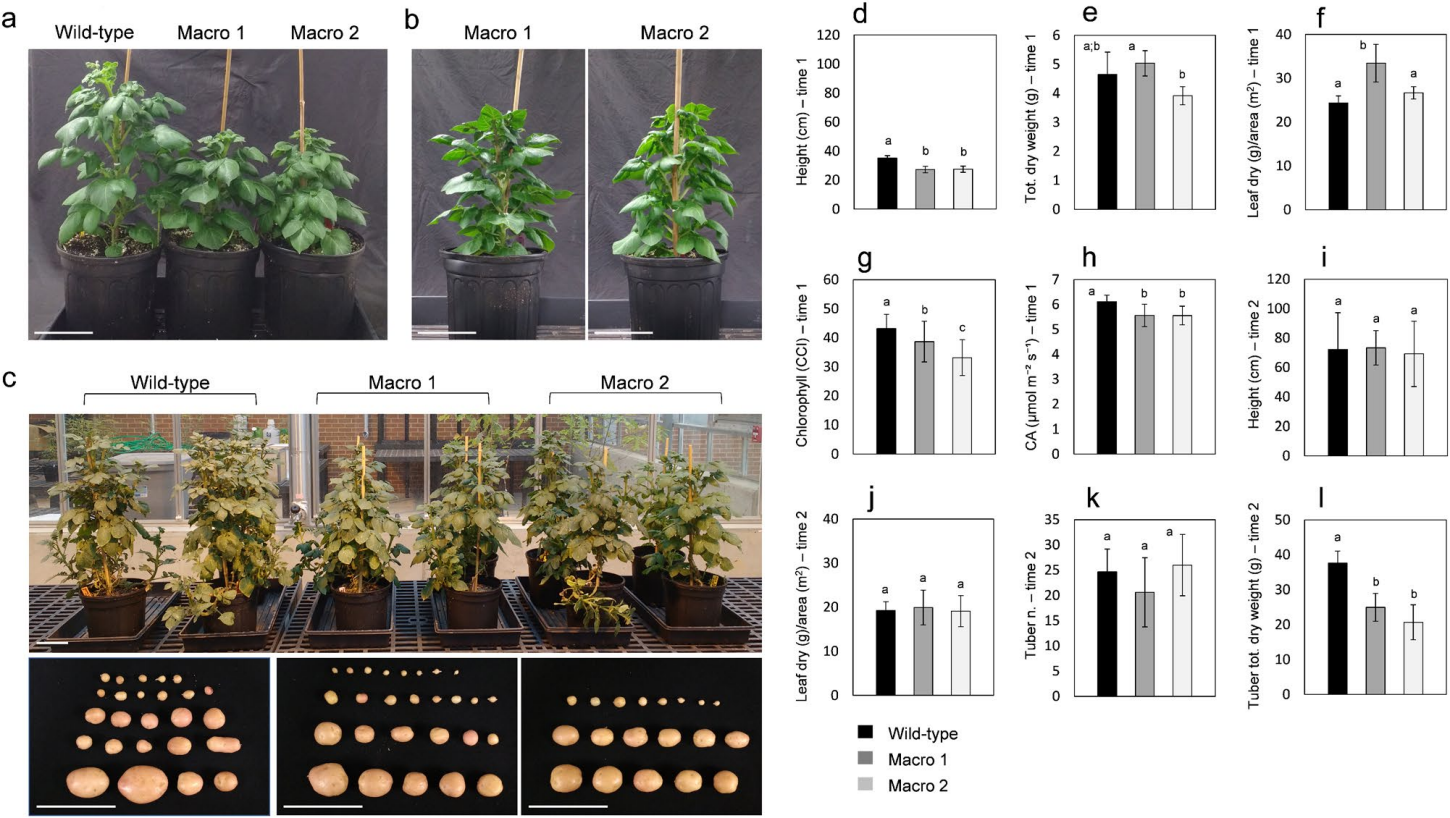
Growth characteristics of *AtFtsZ1* over-expressing potato lines. (**a**) Images of 9-week-old wild-type potato plants and two independent *AtFtsZ1* lines (Macro lines 1 and 2) grown in pots. At 9 weeks, wild-type plants reached anthesis, while macro-chloroplast lines reached the same stage at 10 weeks (1 week later, **b**). (**c**) Images showing 12-week-old wild-type potato plants and macro-chloroplast lines grown in pots. Tubers collected from the same lines grown at the end of life cycle (24-week-old) are also shown. Histograms represent various plant characteristics at anthesis (time 1; **d**–**h**) and at the end of plant life cycle (time 2; **i**–**l**): (**d**,**i**) height; (**e**) total dry weight; (**f**,**j**) ratio of leaf dry weight to foliar area; (**g**) chlorophyll content index (CCI); (**h**) leaf CO_2_ assimilation (CA); (**k**) number of tubers; (**g**) total dry weight of tubers. The results are expressed as mean ± standard deviation of six (time 1) and four (time 2) plants per each genotype. Means were compared using ANOVA and when significant, mean separations were analyzed using Tukey HSD (p < 0.05). Statistical significance is indicated by letters (**a**–**c**). Scale bars: 10 cm (**a**–**c**).

Phenotypic analysis of macro-chloroplast lines compared to wild-type controls was investigated in controlled growth studies with time-point analysis conducted at anthesis and at harvest (Fig. 3). Macro-chloroplast lines demonstrated a 1 week delay in reaching anthesis when compared to wild-type controls (Fig. 3a,b, respectively). Further, at anthesis, the two macro-chloroplast lines were ~ 22% shorter (Fig. 3d) than wild-type controls. Despite this decreased growth, neither line had a decrease in total dry weight when compared to the wild-type control (Fig. 3e and Supplementary Fig. S3a). Despite similar reduction in height between the macro-chloroplast lines, line 1 had significantly more total dry and fresh weight per plant compared to line 2 alone (Fig. 3e and Supplementary Fig. S3a). For both macro-chloroplast lines, chlorophyll content and CO_2_ assimilation were significantly lower than the wild-type lines (Fig. 3g,h, respectively). Reduction in these performance metrics was expected to decrease tuber production due to a lack of available carbon. To evaluate this hypothesis, growth parameters and tuber yield were analyzed at harvest (24-weeks). Images of 12-weeks-old plants (prior to senescence) and tubers harvested at 24-weeks are shown (Fig. 3c). At the end of the life cycle, prior to harvest, the total above-ground biomass is severely affected by extended leaf senescence, thus only plant height and both dry and fresh weight per unit of leaf area were collected. No statistical difference was observed for these growth parameters when comparing macro-chloroplast and wild-type lines (Fig. 3i, j and Supplementary Fig. S4a). Tuber production between individuals was highly variable, leading to no significant difference in tuber number between macro-chloroplast and wild-type lines (Fig. 3k); however, there was a significant reduction in tuber biomass from macro-chloroplast lines (Fig. 3l and Supplementary Fig. S4b). A reduction of ~ 34% and 45% in total dry and a reduction of ~ 31% and 41% in total fresh weight were observed in macro-chloroplast line 1 and 2, respectively (Fig. 3l and Supplementary Fig. S4b).

### Biolistic transformation of chloroplast genomes in macro-chloroplast lines

**Figure 4:**
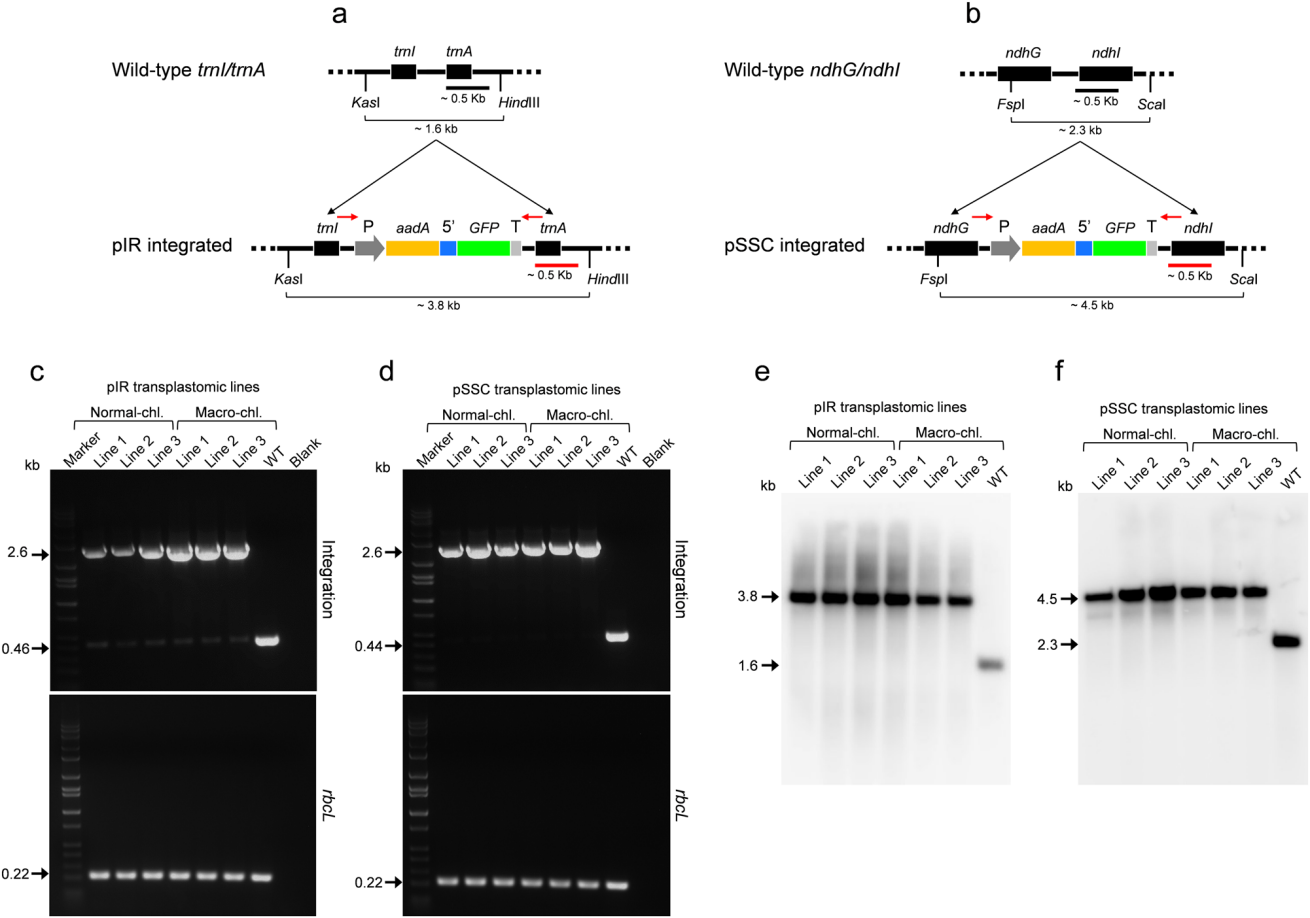
Genotyping of second round of transplastomic lines. Schematic representation of pIR and pSSC vectors (**a**,**b**) integrated into *trnI/trnA* site of the inverted repeat region and *ndhG/ndhI* site of the short single-copy regions of the plastome, respectively. The dual marker operon construct is identical between vectors and includes: *rrn* promoter along with a synthetic RBS (P); *aadA* gene for spectinomycin selection; 5′ untranslated region (5′); a GFP reporter gene; and a terminator/ 3′ untranslated region (T). Restriction enzymes used for Southern blots of pIR and pSSC lines (*Kas*I/*Hind*III and *Fsp*I/*Sca*I, respectively) along with the sizes of expected DNA fragments detected by a ~ 0.5 kb probe (red bar) are indicated. Oligonucleotide used to check integration into either *trnI/trnA* or *ndhG/ndhI* site are indicated (red arrows). Pairs of primers specific for the *trnI/trnA* or *ndhG/ndhI* integration sites were used to check integration in pIR and pSSC lines, respectively (c and d, respectively). Three lines (1–3) for each construct (pIR or pSSC) and genotype (normal or macro) were tested (more lines are shown in Supplementary Fig S5 and S6). DNA bands of 2.6 kb indicate correct integration of both constructs, whereas 0.46 and 0.44 kb-bands indicate the presence of wild-type *trnI/trnA* or *ndhG/ndhI* integration sites, respectively. PCRs specific for the *rbcL* gene (0.22 kb) were used as loading controls. Wild-type samples, blanks and molecular-weight markers are also shown in the gels. Southern blot analysis of genomic DNA from the same pIR and pSSC lines (normal and macro) along with wild-type controls (WT) digested with the indicated restriction enzymes (**a**,**b**) are shown in (**e**) and (**f**), respectively. DNA fragments at 3.8 kb and 4.5 kb indicate the presence of the cassette integrated into either the *trnI/trnA* or *ndhG/ndhI* site of plastome in pIR and pSSC lines, respectively, were fragments at 1.6 kb and 2.3 kb indicate the presence of wild-type DNA only in wild-type controls.

The possibility to transform macro-chloroplasts was investigated by using chloroplast transformation vectors delivered by biolistics. Two vectors, pIR and pSSC specific for recombination into the IR (*trnI*/*trnA*) or SSC (*ndhG*/*ndhI*) regions of plastome were used (Fig. 4a,b, respectively). Considering that the chloroplast morphology and growth parameters of the two *AtFtsZ1* transgenic lines were similar (Figs. 1, 2, 3), only the macro line 1 was used for biolistic transformation. Wild-type potato leaves were used as positive control for plastome transformation. A total of 25 wild-type and 35 macro pIR transplastomic lines along with 16 wild-type and 18 macro pSSC lines were regenerated, respectively (Supplementary Fig. S5 and S6). In wild-type potato, the transformation efficiency for both constructs was higher than macro-chloroplast lines (Table 1).

**Table 1:** Transformation efficiency of chloroplasts from wild-type potato and macro-chloroplast lines transformed with either pIR or pSSC vector.

Construct	Wild-type chloroplasts			Macro-chloroplasts		
	Plates bombarded	Positive plants	Plants/plates bombarded	Plates bombarded	Positive plants	Plants/plates bombarded
pIR	18	64	3.5	26	23	0.9
pSSC	20	78	3.9	26	28	1.1

The number of plates bombarded, the total number of positive plants and the number of plants per each plate bombarded are indicated.

Primers specific for the *trnI*/*trnA* or *ndhG*/*ndhI* integration site along with Southern blots were used to check operon integration in the second round of pIR and pSSC transplastomic lines, respectively (Fig. 4c–f, Supplementary Figs. S5 and S6). PCRs specific for the internal plastome control *rbcL* and the two transgenes (*GFP* and *aadA*) were conducted (Fig. 4c,d and Supplementary Fig. S7). The presence of 2.6 kb DNA-bands at the predicted molecular weight indicated correct operon integration in both plastome regions (*trnI*/*trnA* or *ndhG*/*ndhI*) in all wild-type and macro-chloroplast transplastomic lines (Fig. 4c,d). In Southern blot analysis the presence of bands at the predicted molecular weight for the *trnI*/*trnA* and *ndhG*/*ndhI* integration sites with the cassette integrated (3.8 kb and 4.5 kb, respectively Fig. 4e,f) confirmed correct integration. In Southern blots for both pIR and pSSC lines, the absence of wild-type bands (1.6 kb and 2.3 kb, respectively, Fig. 4e,f) indicates 100% homoplasmy. Compared to normal and macro-chloroplast transplastomic lines, there was no difference in the level of homoplasmy for either integration site (*trnI*/*trnA* or *ndhG*/*ndhI*, Fig. 4e,f).

### GFP accumulation in macro-chloroplast lines

**Figure 5:**
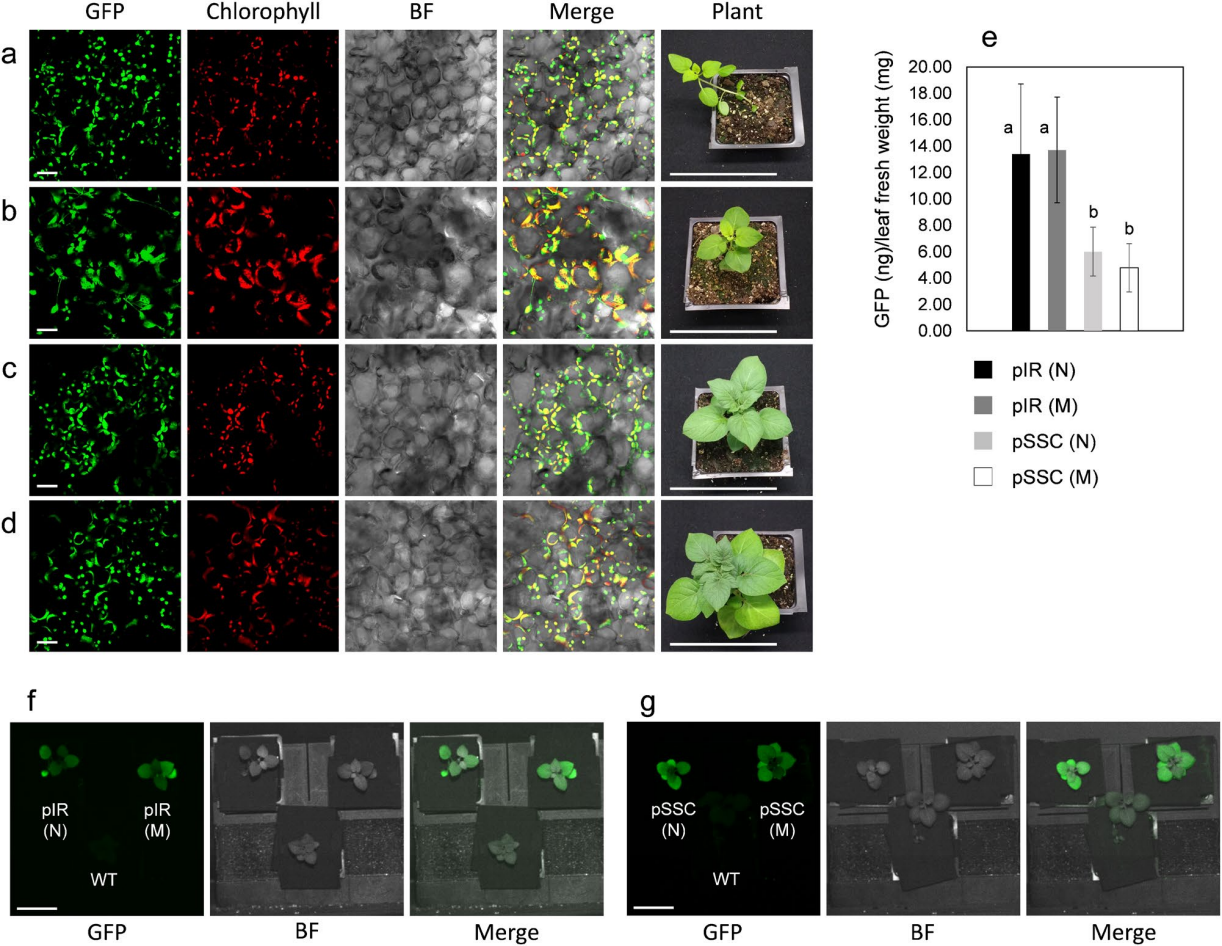
GFP accumulation into chloroplasts of normal transplastomic and macro-chloroplast lines. Confocal images showing GFP localization into the chloroplast stroma of leaf mesophyll cells from normal and macro transplastomic lines, along with pictures showing 3-weeks-old plants grown in pots are indicated: Normal pIR (**a**); macro-chloroplasts pIR (**b**); normal pSSC (**c**); macro-chloroplasts pSSC (**d**). GFP (green), chlorophyll (red), bright field (BF, gray) and merge images (GFP/chlorophyll/bright field) are indicated. Per each genotype (**a**–**d**), images of indicated transplastomic plants are shown. Scale bars: 20 µm (a-d, in GFP, chlorophyll, BF and merge); 10 cm (in plants **a**–**d**). (u) Graph indicating the GFP amount (ng) per unit of leaf fresh weight (mg). Normal (N) and macro-chloroplast (M) lines transformed with either pIR or pSSC constructs are indicated. The results are expressed as mean ± standard deviation (SD) of 2 biological replicate and 2 technical replicate per biological replicate per each independent line. A number of 15 pIR and 8 pSSC lines were used per each genotype, respectively (*N* normal; *M* macro). Means were compared using ANOVA and when significant, mean separations were analyzed using Tukey HSD (p < 0.05). Statistical significance is indicated by numerical letter (**a**,**b**). Images acquired using the FILP (Fluorescence-Inducing Laser Projector) stand-off detection system of transplastomic lines along with wild-type controls (WT) are shown: 3-week-old pIR and pSSC lines of both genotypes (N and M) on potting are shown in f and g, respectively. GFP signal, bright field (BF) and merge images are indicated in (**f**) and (**g**). The images were acquired at 150 ms exposure time. Scale bar: 50 mm (**f**,**g**).

GFP fluorescence was detected using the confocal microscope in chloroplasts in wild-type and macro-chloroplast lines transformed with pIR or pSSC constructs. These fluorescent profiles confirmed GFP production and correct subcellular localization in all transplastomic lines (Fig. 5a–d). The GFP amount produced in leaf tissue was also investigated by fluorometry (Fig. 5e). Quantification of GFP indicated that both normal and macro-chloroplast transplastomic lines accumulated the same amount of protein (ng of GFP) per mg of leaf fresh weight (Fig. 5e). No statistically significant difference was observed in GFP accumulation in both genotypes when compared to transplastomic plants transformed with the same construct, pIR or pSSC. Considering the IR region is present in two copies in the chloroplast genome, both wild-type and macro-chloroplast transplastomic lines transformed with pIR accumulated more protein then pSSC plants (~ 128 and 186% increase, in normal and macro, respectively). In order to investigate if these levels of GFP expression were enough for efficient standoff detection, transplastomic pIR and pSSC lines of both genotypes were imaged using the recently developed FILP (Fluorescence-Inducing Laser Projector) system^43^ (Fig. 5f,g). Fluorescent images of normal and macro-chloroplast lines transformed with pIR or pSSC indicated bright green fluorescence detected in engineered plant canopies at a 3-m standoff (Fig. 5f,g, respectively).

The growth of wild-type and macro-chloroplast transplastomic lines was investigated in real-time for 9 weeks on soil (Supplementary Video S1). As expected, the transplastomic pSSC lines had faster growth compared to the macro-transplastomic pSSC lines. After 9 weeks the transplastomic pSSC lines reached a similar size to wild-type (untransformed) controls, whereas macro-chloroplast lines had reduced height. On the contrary, both transplastomic and macro-transplastomic pIR lines had significantly reduced growth due to the high output of heterologous protein production (Fig. 5e).

## Discussion

The ultrastructural characterization of leaf tissue from potato *AtFtsZ1* lines using TEM showed the presence of large and elongated chloroplasts throughout all analyzed cells (Fig. 1, 2 and Supplementary Figs. S1, S2). This is in accordance with previous results demonstrating that the overexpression of *FtsZ1* affects the normal formation of the z-ring, resulting in inhibition of division and excessive chloroplast growth^36,38,42^. Analyzing TEM micrographs, we did not observe any evidence for ultrastructural differences of thylakoid membranes in macro-chloroplasts compared to wild-type organelles (Fig. 1 and Supplementary Fig. S2).

The results from this work suggest that macro-chloroplast lines have a delay in growth and significant reduction in tuber biomass compared to wild-type controls (Fig. 3 and Supplementary Fig. S3). Despite slower growth, the macro-chloroplast plants had no apparent changes in leaf and stem morphology compared to wild-type controls (Fig. 3). The delay in growth was likely due to reduced photosynthetic performance as supported by reduced amount of chlorophyll and CO_2_ assimilation in macro-chloroplast lines (Fig. 3g,h). Altered growth characteristics may also be the result of altered leaf optical properties in macro-chloroplast lines. It has been demonstrated in tobacco *AtFtsZ* overexpressing lines, the particular chloroplast phenotype and cell rearrangement can lead to either decreased light absorbance (at low-light irradiation) or photodamage of the photosynthetic machinery (at high-light irradiation), resulting in reduced plant growth in both cases^44^.

The prospect of using agronomically-relevant plants with large organelles is an attractive addition to the plastid biotechnology toolkit. Macro-chloroplasts from *AtFtsZ1* transgenic lines can be transformed using biolistics and transplastomic lines can be regenerated using standard tissue culture. We anticipated that increasing the size of the target organelle may provide an advantage in transformation efficiency in potato as it has been previously demonstrated in tobacco^41^. Considering the transformation efficiencies of potato chloroplasts reported in the literature^45–47^, our macro-chloroplast lines can be efficiently transformed, yielding approximately 1 transplastomic plant per each plate bombarded (0.9 and 1.1 for pIR and pSSC construct, respectively, Table 1). However, the transformation efficiency of transgenic lines was not greater than the efficiency obtained in our laboratory for wild-type potato leaves. For both constructs tested (pIR and pSSC) about 4 positive plants per each plate bombarded (3.5 and 3.9, respectively, Table 1) were obtained. This observation suggests that there is not a clear advantage of having larger organelles using biolistics. This low transformation efficiency could be due to the particular spatial distribution of macro-chloroplasts in leaf mesophyll cells. Compared to wild-type, macro-chloroplasts seem not to be regularly distributed throughout the cytoplasm and are instead pushed to the plasma membrane (Fig. 1d–f). This particular distribution may reduce the probability of gold particles hitting the target organelle.

Nevertheless, this result does not exclude the potential for macro-chloroplasts to be desirable targets for other gene-delivery methods in which the transformable surface is an important limiting factor. For almost 3 decades, the gene gun has been the most efficient and reproducible method used for chloroplast transformation^48,49^. This is an invasive physical method that could induce the shearing of large vectors and, thus, is not particularly suited for transformation of large, chromosome-sized constructs. The effective transformation of large DNA molecules, such as an entire synthetic plastome, may necessitate alternative methods of transformation. Many methods including micro-injection^50,51^, polyethylene glycol (PEG) transformation^52,53^ and electroporation^54,55^ have been used to transform chloroplasts. Different kinds of commercial carbon-nanotubes have also been used to deliver DNA into plants cells^56,57^, and these delivery techniques could also represent an alternative to deliver vectors more efficiently into macro-chloroplasts. Thus, macro-chloroplasts could provide considerable advantages in increasing transformation efficiency using these methods.

Compared to conventional transplastomic lines, macro-chloroplast lines do not differ in the level of homoplasmy for both *trnI*/*trnA* and *ndhG*/*ndhI* integration sites (Fig. 4). Two rounds of selection in tissue culture were required to get pIR and pSSC homoplasmic lines (*trnI*/*trnA* and *ndhG*/*ndhI*) in both macro- and wild-type chloroplast-engineered plants. While a difference in plastome copy number was previously observed in macro-chloroplast and wild-type tobacco^58^, our results suggest that potato *AtFtsZ1* overexpressing lines have similar plastome copy number (Fig. 1c). Further, similar heterologous protein (GFP) production was observed in wild-type and macro-transplastomic lines and varied by construct/integration site, as expected (Fig. 5e). Based on growth analysis of pSSC transplastomic and macro-transplastomic lines, both reached full maturity, while pIR transplastomic and macro-transplastomic lines demonstrated significantly stunted growth due to the increased production of heterologous protein. These results suggest that the amount of heterologous protein production is a much more significant consideration than chloroplast size when conducting chloroplast biotechnology. Moreover, as observed for wild-type chloroplasts, the level of GFP accumulation obtained in macro-chloroplast lines was enough for efficient standoff imaging of whole plant canopy using the FILP system (Fig. 5f,g). This is of particular interest in a context of modern agriculture, where phenomics has the prospect to bring together high-dimensional phenotypic and molecular data together with their interaction with environmental stimuli^43,59^.

## Methods

### Plant growth conditions and phenotypic analysis

*Solanum tuberosum* (potato) var. ‘Desirée’ and *AtFtsZ1* overexpressing lines were grown in Magenta boxes containing MS Reg media (4.33 g/l MS basal salt mixture; 25 g/l sucrose; 100 mg/l myo-inositol; 170 mg/l sodium phosphate monobasic monohydrate; 440 mg/l calcium chloride dihydrate; 0.9 mg/l thiamine-HCl; 2 mg/l glycine; 0.5 mg/l nicotinic acid; 0.5 mg/l pyridoxine–HCl; 1 × MS vitamins; 3 g/l phytagel; pH 5.8). Transplastomic lines of both genotypes (wild-type and macro) were grown in selective MS rooting media^60^.

For determination of growth characteristics, apical shoots (same length and age) from both transgenic and wild-type plants were in vitro propagated. After 2 weeks, small plantlets with roots were transferred to soil, Pro-Mix BK25 (Griffin Greenhouse Supplies, Inc. Tewksbury, MA, USA), and grown to anthesis (9- and 10-weeks-old for wild-type and *AtFtsZ1* lines, respectively) or full maturity (24-weeks-old for both genotypes). Pots of 3.8 or 11.4 l volume were used to grow plants at anthesis or full maturity, respectively. Plant height (cm), fresh and dry weight (g) of leaves and total biomass were measured. The total number, along with the dry and fresh weights (g) of tubers were also measured. For determination of dry weight, plant tissue was dried for 1 week at 50 °C. Both leaf and tuber areas were obtained by image analysis using ImageJ 1.41o software (National Institute of Health, Bethesda, MD, USA).

CO_2_ assimilation (A) values per unit of leaf area (µmol m^−2^ s^−1^) were obtained using a LI-6800 portable photosynthesis system (LI–COR Biosciences, Lincoln, NE, USA) at atmospheric CO_2_ concentrations (400 µmol mol air^−1^), constant irradiance (1000 µmol photons m^−2^ s^−1^), ambient temperature of 23 °C, a vapour pressure deficit (VPD leaf) of 0.8–1.2 kPa and a flow rate of 200 µmol s^−1^). The leaf chlorophyll content index (CCI) was obtained using a portable CCM-200 plus chlorophyll content meter (OPTI-SCIENCES Inc., Hudson, NH, USA).

Results were expressed as mean ± standard deviation (sd) of six and four biological replicates for each genotype (*AtFtsZ1* independent lines (Macro 1 and 2) along with wild-type control) for the growth experiments at anthesis and full maturity, respectively. ANOVAs with post-hoc Tukey statistical analysis (IBM SPSS software) (p < 0.05) were performed to determine if differences were statistically significant among wild-type and *AtFtsZ1* lines. Plants grown in vitro and in pots were subjected to congruent environmental conditions, 16/8 h of light and dark respectively, and the temperature was kept constant at 24 °C.

### Construction of transformation vectors

The vector pAP202 containing the full-length cDNA of *AtFtsZ1* was used to generated *AtFtsZ1* overexpressing lines as published previously^36^. For the construction of pIR and pSSC, the tobacco (*Nicotiana tabacum*) plastome IR (*trnI/trnA*; 102,623–105,457 bp and 105,458–110,067 bp; GenBank: KU199713.1) and SSC (*ndhG/ndhI*; 119,184–120,988 bp and 120,989–126,029 bp; GenBank: KU199713.1) regions, respectively, were synthesized by GeneArt (Thermo Fisher Scientific, Waltham, MA, USA). The two homologous regions were modified including several SNPs to facilitate cloning. Before introducing the selection cassette between homologous arms, the *trnI/trnA* and *ndhG/ndhI* regions were cloned into pMK vector (Thermo Fisher Scientific, Waltham, MA, USA). A chloroplast selection cassette (*Prrn-SD::aadA::5′UTR::GFP::psbA3′UTR*) was PCR amplified from the pLD-PTD-GFP plasmid^61^ using 1Fw/1Rv primers and cloned into the *Pme*I site of *trnI/trnA* and *ndhG/ndhI* sites generating pIR and pSSC plasmids, respectively. A list of primers used in this work is shown in Supplementary Table S1.

### Generation of transgenic lines

Potato *AtFtsZ1* overexpressing lines (macro-chloroplast lines) were generated by *Agrobacterium*-mediated transformation. The vector pAP202^36^ was introduced into *Agrobacterium tumefaciens* LBA4404 by the freeze thaw method^62^. For each transformation, 20 internodes of ~ 1 cm-length from 1-month-old in vitro potato plants were used. Internodes were then transformed via *Agrobacterium* and transgenic plants were regenerated in selective media, as previously described^63^. Putative transgenic lines were screened by fluorescent microscopy for clearly-defined enlarged chloroplasts. Thereafter, selected lines were genetically characterized for construct integration and transgene expression.

The PDS-1000/He biolistics device (Bio-Rad, Hercules, CA, USA) was subsequently used to transform chloroplasts of both macro-chloroplast and wild-type potato plants^64^. Macro-chloroplast line 1 was used for chloroplast transformation. Per each transformation ~ 6 cm^2^ of leaf tissue from 1-month-old in vitro potato plants were used. Per each shoot 0.3 mg of gold-particles (0.6 µm in diameter) binding 1 µg of plasmid were used following the manufacturer protocol (Seashell Technology, La Jolla, CA, USA). Transplastomic plants of both genotypes (wild-type and macro-chloroplast) were then regenerated from transformed leaf material incubated in selective media as described previously^60^. The second round of transplastomic plants were obtained by applying the same protocol of tissue culture/selection/regeneration^60^. All lines were genetically characterized for the presence of the selection cassette integrated and GFP expression.

### Total DNA extraction and PCR analysis

Total genomic-DNA preparations from leaves were obtained using ~ 50 mg of tissue and the CTAB-based procedure of extraction^64^. PCR (25 cycles) was performed in 25 µl reaction-volume by using 5 ng of total genomic DNA and the DreamTaq Green PCR Master Mix (Thermo Fisher Scientific, Waltham, MA, USA). For molecular characterization of *AtFtsZ1* overexpressing lines, the pairs of primers 2Fw/2Rv and 3Fw/3Rv were used to check for *nptII/AtFtsZ1* cassette integration and the internal control *actin* (GeneID-102593904), respectively. For genotyping of transplastomic lines, the two pairs of primers 4Fw/4Rv and 5Fw/5Rv were used to verify cassette integration in the *trnI/trnA* and *ndhG/ndhI* sites of plastome, respectively. The two pair of primers 6Fw/6Rv and 7Fw/7Rv were used to amplify full-length *GFP* (NCBI ID: AEX93343.1) and *aadA* (NCBI ID: AAR14532.1) genes, respectively. The primers 8Fw/8Rv were used to amplify an internal portion of *rbcL* (NCBI ID: 4099985).

### Total RNA extraction and reverse transcriptase PCR

Total RNA extraction was performed using Tri-Reagent (Molecular Research Center, Inc, Cincinnati, OH, USA) according to manufacturer’s protocol. Per each extraction ~ 50 mg healthy leaf tissue was used. Total RNA preparations were cleaned and subjected to DNase treatment using the RNA Clean & Concentrator Kit (Zymogen, Irvine, CA, USA) according to manufacturer’s instruction. The cDNA synthesis was performed using the Super Script III Reverse Transcriptase (Thermo Fisher Scientific, Waltham, MA, USA) following the manufacturer’s instruction. cDNA was quantified using a NanoDrop (Thermo Fisher Scientific, Waltham, MA, USA), and several serial cDNA dilutions were tested by PCR (as described before) for the presence of the transgene *AtFtsZ*, whereas the two internal controls *rbcL* (plastome) and *ef1α* (nuclear) were used for normalization. The pair of primers 9Fw/9Rv, 10Fw/10Rv and 11Fw/11Rv were used to amplify and internal fragment (~ 100 bp) of *AtFtsZ* (Tair ID: AT5G55280), *rbcL* (NCBI ID: 4,099,985) and *ef1α* (NCBI ID: NM_001288491.1), respectively.

### Southern blot analysis

DNA probes for detection of pIR and pSSC constructs integrated into potato plastome (GenBank: NC_008096.2) were designed on IR (104,457–104,978 bp) and SSC (120,269–120,790 bp) regions, respectively. The PCR DIG Probe Synthesis Kit (Roche, Indianapolis, IN, USA) was used to synthesize digoxigenin(DIG)-sUTP-labelled IR and SSC DNA-probes using the pair of primers 12Fw/12Rv and 13Fw/13Rv, respectively. Total genomic DNA from leaf tissue was extracted using CTAB, as described above. After quantification, 1 µg of DNA for each pIR and pSSC sample was digested using *Kas*I/*Hind*III and *Fsp*I/*Sca*I restriction enzymes, respectively. The DNA fragments were separated on 0.9% agarose gel, and after that, the gel was depurinated, denatured and transferred on a nylon membrane as described previously^13^. The membrane was then incubated with the DIG-labelled probe and detected using the anti- digoxigenin-AP Fab fragments detection kit (Roche Indianapolis, IN, USA) accordingly to the manufacturer’s protocol.

### Real-Time PCR

PCRs were performed in 96-well plates (Thermo Fisher Scientific, Waltham, MA, USA), in a total volume of 15 µl per reaction using 1X PowerUp SYBR Green Master Mix (Thermo Fisher Scientific, Waltham, MA, USA), 5 ng of total genomic DNA from leaves and 0.5 µM of each primer. Primers 20 bp-long with an annealing temperature of ~ 57 °C and able to amplify a ~ 100 bp-fragment were design using the online software Primer3 input v. 0.4.0 (Howard Hughes Medical Institute and by the National Institutes of Health)^65^. The primers 10Fw/10Rv and 14Fw/14Rv were use to detect the plastome gene *rbcL* (NCBI ID: 4099985) and the nuclear gene *actin* (GeneID-102593904), respectively (Supplementary Table S1). The QuantStudio 6 Flex Real-Time PCR System (Thermo Fisher Scientific, Waltham, MA, USA) was used to perform real-time PCR, whereas the QuantStudio Real-Time PCR Software v1.1 (Thermo Fisher Scientific, Waltham, MA, USA) was used to aquire amplification data. The data were expressed as Log_2_(2^−ΔCT^) of *rbcL* vs *actin*. Results were expressed as mean ± standard deviation (sd) of 3 biological replicates and 8 technical replicates per biological replicate per each genotype. An ANOVA post-hoc Tukey statistical analysis (p < 0.05) was performed to determine statistically significant difference among means (IBM SPSS software).

### GFP quantification

The amount of GFP accumulated in leaf tissue of transplastomic lines was quantified by using the Fluorometric GFP Quantification Kit (Cell Biolabs, Inc., San Diego, CA, USA) according to the manufacturer’s protocol^66^. The results were expressed as mean ± standard deviation (ng GFP/mg fresh weight) of two indipendent experiments, resulting in a total of 2 biological replicates and 2 technical replicates per biological replicate for each independent transplastomic line. We analyzed 15 pIR and 8 pSSC lines for each genotype, normal and macro-chloroplast.

### Confocal microscopy and chloroplast size determination

Leaf tissue from 3-week-old in vitro plants was imaged using an Olympus Fv1200 confocal microscope (Olympus, Center Valley, PA, USA). GFP was excited at 488 nm and detected at an emission wavelength of 509 nm. Chlorophyll autofluorescence was visualized using an excitation wavelength of 543 nm and an emission wavelength of 667 nm. Confocal images were acquired using the manufacturer’s Olympus FV10-ASW Viewer software Ver.4.2a (Olympus, Center Valley, PA). Confocal images were processed using the online software ImageJ 1.41o (National Institute of Health, Bethesda, MD, USA). The same ImageJ software was also used to estimate chloroplast size in both *AtFtsZ1* overexpressing lines and wild-type controls. The results were expressed as mean ± standard deviation (sd) and the statistical analysis was performed using SPSS statistics 25 software (IBM).

### Transmission electron microscope (TEM)

Leaf tissue from 3-week-old in vitro plants were chemically fixed in glutaraldehyde/ paraformaldehyde along with an osmium tetroxide solution and embedded in EMBed-821 resin (Electron Microscopy Sciences, Hatfield, PA, USA) as described previously^67^. Ultrathin sections (60–100 nm) of embedded tissue were cut using a diamond knife and a Leica EM UC7 ultramicrotome (Leica, Buffalo Grove, IL, USA). Ultrathin sections were post strained using aqueous solutions of uranyl acetate and lead citrate as described previously^67^. Images were obtained using a JEOL 1400 transmission electron microscope operating at 80 kV (JEOL, Peabody, MA, USA) equipped with a Gatan OneView camera (Gatan, Pleasanton, CA, USA). TEM micrographs were processed using the software ImageJ 1.41o (National Institute of Health, Bethesda, MD, USA).

### Fluorescence imaging by the fluorescence-inducing laser projector (FILP)

Plant fluorescence patterns were characterized using the Fluorescence-Inducing Laser Projector (FILP), a custom-built instrument for imaging fluorescence in plants. FILP imaging was performed on 3-week-old transplastomic lines in pots^43^. GFP fluorescence in plants was acquired at 150 ms exposure time using 465 nm excitation and 525 nm emission notch (50 nm) filter. Standoff detection was performed at 3 m from the laser source. FILP images were processed using ImageJ 1.41o software (National Institute of Health, Bethesda, MD, USA).

## Supplementary information

Supplementary Information 1.

Supplementary Video 1.

## References

[1] Bock R (2015). Engineering plastid genomes: methods, tools, and applications in basic research and biotechnology. Annu Rev Plant Biol.

[2] Jin S, Daniell H (2015). The engineered chloroplast genome just got smarter. Trends Plant Sci..

[3] Maliga P, Bock R (2011). Plastid biotechnology: food, fuel, and medicine for the 21st century. Plant Physiol..

[4] Wurbs D, Ruf S, Bock R (2007). Contained metabolic engineering in tomatoes by expression of carotenoid biosynthesis genes from the plastid genome. Plant J..

[5] Pasoreck EK (2016). Terpene metabolic engineering via nuclear or chloroplast genomes profoundly and globally impacts off-target pathways through metabolite signalling. Plant Biotechnol. J..

[6] Daniell H, Rai V, Xiao Y (2019). Cold chain and virus-free oral polio booster vaccine made in lettuce chloroplasts confers protection against all three poliovirus serotypes. Plant Biotechnol. J..

[7] Daniell H, Kulis M, Herzog RW (2019). Plant cell-made protein antigens for induction of oral tolerance. Biotechnol. Adv..

[8] Daniell H (2020). Investigational new drug enabling angiotensin oral-delivery studies to attenuate pulmonary hypertension. Biomaterials.

[9] Park J (2020). Oral delivery of novel human IGF-1 bioencapsulated in lettuce cells promotes musculoskeletal cell proliferation, differentiation and diabetic fracture healing. Biomaterials.

[10] Daniell H (2019). Validation of leaf and microbial pectinases: commercial launching of a new platform technology. Plant Biotechnol. J..

[11] Kumari U (2019). Validation of leaf enzymes in the detergent and textile industries: launching of a new platform technology. Plant Biotechnol. J..

[12] Lin MT, Occhialini A, Andralojc PJ, Parry MA, Hanson MR (2014). A faster Rubisco with potential to increase photosynthesis in crops. Nature.

[13] Occhialini A, Lin MT, Andralojc PJ, Hanson MR, Parry MA (2016). Transgenic tobacco plants with improved cyanobacterial Rubisco expression but no extra assembly factors grow at near wild-type rates if provided with elevated CO_2_. Plant J..

[14] Chen PJ (2014). Transplastomic *Nicotiana benthamiana* plants expressing multiple defence genes encoding protease inhibitors and chitinase display broad-spectrum resistance against insects, pathogens and abiotic stresses. Plant Biotechnol. J..

[15] Jin S, Kanagaraj A, Verma D, Lange T, Daniell H (2011). Release of hormones from conjugates: chloroplast expression of β-glucosidase results in elevated phytohormone levels associated with significant increase in biomass and protection from aphids or whiteflies conferred by sucrose esters. Plant Physiol..

[16] Arlen PA (2007). Field production and functional evaluation of chloroplast-derived interferon-α2b. Plant Biotechnol. J..

[17] Daniell H (2007). Transgene containment by maternal inheritance: effective or elusive?. Proc. Natl. Acad. Sci. USA.

[18] Schindel HS, Piatek AA, Stewart CN, Lenaghan SC (2018). The plastid genome as a chassis for synthetic biology-enabled metabolic engineering: players in gene expression. Plant Cell Rep..

[19] Valkov VT (2009). Genome-wide analysis of plastid gene expression in potato leaf chloroplasts and tuber amyloplasts: transcriptional and posttranscriptional control. Plant Physiol..

[20] Verma D, Daniell H (2007). Chloroplast vector systems for biotechnology applications. Plant Physiol..

[21] Bock R (2013). Strategies for metabolic pathway engineering with multiple transgenes. Plant Mol. Biol..

[22] Lu Y, Rijzaani H, Karcher D, Ruf S, Bock R (2013). Efficient metabolic pathway engineering in transgenic tobacco and tomato plastids with synthetic multigene operons. Proc. Natl. Acad. Sci. USA.

[23] Ruf S (2019). High-efficiency generation of fertile transplastomic Arabidopsis plants. Nat. Plants.

[24] Fuentes P (2016). A new synthetic biology approach allows transfer of an entire metabolic pathway from a medicinal plant to a biomass crop. eLife.

[25] Su J (2015). Low cost industrial production of coagulation factor IX bioencapsulated in lettuce cells for oral tolerance induction in hemophilia B. Biomaterials.

[26] Yu Q, Barkan A, Maliga P (2019). Engineered RNA-binding protein for transgene activation in non-green plastids. Nat. Plants.

[27] Lee SM (2006). Plastid transformation in the monocotyledonous cereal crop, rice (*Oryza sativ*a) and transmission of transgenes to their progeny. Mol. Cells.

[28] Wang Y, Wei Z, Xing S (2018). Stable plastid transformation of rice, a monocot cereal crop. Biochem. Biophys. Res. Commun..

[29] Langbecker CL (2004). High-frequency transformation of undeveloped plastids in tobacco suspension cells. Plant Physiol..

[30] Osteryoung KW, Stokes KD, Rutherford SM, Percival AL, Lee WY (1998). Chloroplast division in higher plants requires members of two functionally divergent gene families with homology to bacterial *ftsZ*. Plant Cell.

[31] Schmitz AJ, Glynn JM, Olson BJ, Stokes KD, Osteryoung KW (2009). Arabidopsis FtsZ2-1 and FtsZ2-2 are functionally redundant, but FtsZ-based plastid division is not essential for chloroplast partitioning or plant growth and development. Mol. Plant.

[32] McAndrew RS, Froehlich JE, Vitha S, Stokes KD, Osteryoung KW (2001). Colocalization of plastid division proteins in the chloroplast stromal compartment establishes a new functional relationship between FtsZ1 and FtsZ2 in higher plants. Plant Physiol..

[33] Mori T, Kuroiwa H, Takahara M, Miyagishima SY, Kuroiwa T (2001). Visualization of an FtsZ ring in chloroplasts of *Lilium longiflorum* leaves. Plant Cell Physiol..

[34] Vitha S, McAndrew RS, Osteryoung KW (2001). FtsZ ring formation at the chloroplast division site in plants. J. Cell Biol..

[35] Chen C, MacCready JS, Ducat DC, Osteryoung KW (2018). The molecular machinery of chloroplast division. Plant Physiol..

[36] Stokes KD, McAndrew RS, Figueroa R, Vitha S, Osteryoung KW (2000). Chloroplast division and morphology are differentially affected by overexpression of *FtsZ1* and *FtsZ2* genes in Arabidopsis. Plant Physiol..

[37] Jeong WJ, Jeong SW, Min SR, Yoo OJ, Liu JR (2002). Growth retardation of plants transformed by overexpression of *NtFtsZ1-2* in tobacco. J. Plant Biol..

[38] de Pater S (2006). Manipulation of starch granule size distribution in potato tubers by modulation of plastid division. Plant Biotechnol. J..

[39] Mellor SB, Behrendorff J, Nielsen AZ, Jensen PE, Pribil M (2018). Non-photosynthetic plastids as hosts for metabolic engineering. Essays Biochem..

[40] Ruf S, Hermann M, Berger IJ, Carrer H, Bock R (2001). Stable genetic transformation of tomato plastids and expression of a foreign protein in fruit. Nat. Biotechnol..

[41] Jeong WJ, Min SR, Liu JR (2006). Enhancement of chloroplast transformation frequency by using mesophyll cells containing a few enlarged chloroplasts from nuclear transformed plants in tobacco. J. Plant Biotechnol..

[42] Chikkala VRN, Nugent GD, Stalker DM, Mouradov A, Stevenson TW (2012). Expression of *Brassica oleracea FtsZ1-1* and *MinD* alters chloroplast division in *Nicotiana tabacum* generating macro- and mini-chloroplasts. Plant Cell Rep..

[43] Rigoulot SB (2020). Imaging of multiple fluorescent proteins in canopies enables synthetic biology in plants. Plant Biotechnol. J..

[44] Jeong WJ (2002). A large population of small chloroplasts in tobacco leaf cells allows more effective chloroplast movement than a few enlarged chloroplasts. Plant Physiol..

[45] Nguyen TT, Nugent G, Cardi T, Dix PJ (2005). Generation of homoplasmic plastid transformants of a commercial cultivar of potato (*Solanum tuberosum* L.). Plant Sci..

[46] Sidorov VA (1999). Technical advance: stable chloroplast transformation in potato: use of green fluorescent protein as a plastid marker. Plant J.

[47] Valkov VT (2011). High efficiency plastid transformation in potato and regulation of transgene expression in leaves and tubers by alternative 5' and 3' regulatory sequences. Transgenic Res..

[48] Svab Z, Hajdukiewicz P, Maliga P (1990). Stable transformation of plastids in higher plants. Proc. Natl. Acad. Sci. USA.

[49] Svab Z, Maliga P (1993). High-frequency plastid transformation in tobacco by selection for a chimeric aadA gene. Proc. Natl. Acad. Sci. USA.

[50] Knoblauch M, Hibberd JM, Gray JC, van Bel AJ (1999). A galinstan expansion femtosyringe for microinjection of eukaryotic organelles and prokaryotes. Nat. Biotechnol..

[51] Verhoeven HA, Blaas J (1992). Direct cell to cell transfer of organelles by microinjection. Plant Cell Rep..

[52] Golds T, Maliga P, Koop H-U (1993). Stable plastid transformation in PEG-treated protoplasts of *Nicotiana tabacum*. Bio/Technology.

[53] Koop H-U, Kofer W, Ingo P, German S (1995). Gene Transfer to Plants.

[54] To KY, Cheng MC, Chen LF, Chen SC (1996). Introduction and expression of foreign DNA in isolated spinach chloroplasts by electroporation. Plant J..

[55] Zhang R (2014). High-throughput genotyping of green algal mutants reveals random distribution of mutagenic insertion sites and endonucleolytic cleavage of transforming DNA. Plant Cell.

[56] Yuan H (2011). Single walled carbon nanotubes exhibit dual-phase regulation to exposed Arabidopsis mesophyll cells. Nanoscale Res. Lett..

[57] Kwak SY (2019). Chloroplast-selective gene delivery and expression in planta using chitosan-complexed single-walled carbon nanotube carriers. Nat. Nanotechnol..

[58] Osteryoung KW, Pyke KA (2014). Division and dynamic morphology of plastids. Annu. Rev. Plant. Biol..

[59] Houle D, Govindaraju DR, Omholt S (2010). Phenomics: the next challenge. Nat. Rev. Genet..

[60] Valkov VT, Gargano D, Scotti N, Cardi T (2014). Plastid transformation in potato: *Solanum tuberosum*. Methods Mol. Biol..

[61] Kwon KC, Verma D, Jin S, Singh ND, Daniell H (2013). Release of proteins from intact chloroplasts induced by reactive oxygen species during biotic and abiotic stress. PLoS ONE.

[62] Weigel D, Glazebrook J (2006). Transformation of agrobacterium using the freeze-thaw method. CSH Protoc..

[63] Chronis D (2013). A ubiquitin carboxyl extension protein secreted from a plant-parasitic nematode *Globodera rostochiensis* is cleaved in planta to promote plant parasitism. Plant J.

[64] Occhialini A (2019). MoChlo: a versatile, modular cloning toolbox for chloroplast biotechnology. Plant Physiol.

[65] Untergasser A (2012). Primer3-new capabilities and interfaces. Nucleic Acids Res..

[66] Wamboldt Y (2009). Participation of leaky ribosome scanning in protein dual targeting by alternative translation initiation in higher plants. Plant Cell.

[67] Lin MT (2014). β-Carboxysomal proteins assemble into highly organized structures in Nicotiana chloroplasts. Plant J.

