# Supplementary material for: Generation, analysis, and transformation of macro-chloroplast Potato (Solanum tuberosum) lines for chloroplast biotechnology
Source: Sci Rep. 2020 Dec 3;10:21144. doi: 10.1038/s41598-020-78237-x (PMC7713401; doi:10.1038/s41598-020-78237-x)
Supplement: Supplementary file 1 — Supplementary Information 1. [file 41598_2020_78237_MOESM1_ESM.docx]

**Supplementary Material**

**Generation, analysis, and transformation of macro-chloroplast Potato (*Solanum tuberosum)* lines for chloroplast biotechnology**

Alessandro Occhialini^1,2^, Alexander C. Pfotenhauer^1,2^, Taylor P. Frazier^3,4^, Li Li^2,3^, Stacee A. Harbison^2,3^, Andrew J. Lail^2,3^, Zachary Mebane^3^, Agnieszka A. Piatek^3^, Stephen B. Rigoulot^2,3^, Henry Daniell^5^, C. Neal Stewart, Jr^2,3^, Scott C. Lenaghan^1,2,*^

^1^Department of Food Science, University of Tennessee, Knoxville, Tennessee 37996, USA.

^2^Center for Agricultural Synthetic Biology (CASB), University of Tennessee Institute of Agriculture, Knoxville, Tennessee 37996, USA.

^3^Department of Plant Sciences, University of Tennessee, Knoxville, Tennessee 37996, USA.

^4^Elo Life Systems, Durham, North Carolina 27709, USA.

^5^Department of Biochemistry, School of Dental Medicine, University of Pennsylvania, Philadelphia (PA), USA.

ORCID IDs: 0000-0002-1162-798X (A.O.); 0000-0002-5015-7765 (A.C.P.); 0000-0003-3026-9193 (C.N.S.); 0000-0002-7539-1726 (S.C.L.).

*Correspondence:

Scott C. Lenaghan, Department of Food Science, 102 Food Safety and Processing Building 2600 River Dr., Knoxville TN 37996, USA.

Telephone: 865.974.0098

E-mail: slenagha@utk.edu

**Supplementary Table S1**. Primers used in this study. The primer id, the full name and the nucleotide sequence (from 5’ to 3’) are indicated in the table. The primers are subdivided in forward (1 Fw-14 Fw) and reverse (1 Rv-14 Rv) primers.

| **Primers Forward** | |  |  |  |  |  |  |
| --- | --- | --- | --- | --- | --- | --- | --- |
| **id** | **name** | **sequence (5'-3')** | |  |  |  |  |
| 1 Fw | Selectio-Cassette-1-Fw | CAATGTGAGTTTTTGTAGTTGGATTTGCTCC | | | | |  |
| 2 Fw | nptII/AtFtsZ1-Cass-Fw | TCGGGGTAGGTGTTTCTT | | |  |  |  |
| 3 Fw | Actin-P-Fw | CGGAGCGTGGTTACTCATTT | | | |  |  |
| 4 Fw | trnA-Fw | CAGTAGAGTCTTTCAGTGGCACGTT | | | | |  |
| 5 Fw | SSC2-Fw | CCCCCTAATATAAGACCCGACCC | | | |  |  |
| 6 Fw | mGFP-full-Fw | ATGAGTAAAGGAGAAGAACTTT | | | |  |  |
| 7 Fw | SmR-full-Fw | ATGGCAGAAGCGGTGATC | | |  |  |  |
| 8 Fw | rbcL-P-Fw | GCTGCCGAATCTTCTACTGG | | | |  |  |
| 9 Fw | AtFtsZ-qPCR Fw | AGCGGTTTACAGAGTGTTGA | | | |  |  |
| 10 Fw | rbcL-q-Fw | AGATCTGCGAATCCCTGTTG | | | |  |  |
| 11 Fw | ef1-alpha-q-Fw | ATTGGAAACGGATATGCTCCA | | | |  |  |
| 12 Fw | IR-probe-Fw | GATATAGCTCAGTTGGTAGAGCTCCGCTCT | | | |  |  |
| 13 Fw | SSC-probe-Fw | CAACCACTAGTTTGAATTGCCCAAGCAAAA | | | |  |  |
| 14 Fw | Actin-q-Fw | GCTTCCCGATGGTCAAGTCA | | | |  |  |
| **Primers Reverse** | |  |  |  |  |  |  |
| **id** | **name** | **sequence (5'-3')** | |  |  |  |  |
| 1 Rv | Selectio-Cassette-1-Rv | CTGCAGCCCAAACAAATACAAAATCAAAATAGA | | | | | |
| 2 Rv | nptII/AtFtsZ1-Cass-Rv | TTCAGTGACAACGTCGAGCA | | | |  |  |
| 3 Rv | Actin-P-Rv | GCAGCTTCCATTCCAATCAT | | | |  |  |
| 4 Rv | trnI-Rv | GCCAGGGTAAGGAAGAAGGGG | | | |  |  |
| 5 Rv | SSC2-Rv | CCGAATTACGAAGGCTTAGTTCGG | | | |  |  |
| 6 Rv | mGFP-full-Rv | TTATTTGTATAGTTCATCCATGCC | | | |  |  |
| 7 Rv | SmR-full-Rv | TTATTTGCCGACTACCTTGGT | | | |  |  |
| 8 Rv | rbcL-P-Rv | CAGGGCTTTGAACCCAAATA | | | |  |  |
| 9 Rv | AtFtsZ-qPCR Rv | AGCCCACGAGTTAAAAGTTC | | | |  |  |
| 10 Rv | rbcL-q-Rv | CAGGGGACGACCATACTTGT | | | |  |  |
| 11 Rv | ef1-alpha-q-Rv | TCCTTACCTGAACGCCTGTCA | | | |  |  |
| 12 Rv | IR-probe-Rv | GCGGACAGCTAATGCGTTCCACTTATTGAA | | | |  |  |
| 13 Rv | SSC-probe-Rv | ATGATTACCCTGTCCCACGCAAATCGTTTA | | | |  |  |
| 14 Rv | Actin-q-Rv | GGATTCCAGCTGCTTCCATTC | | | |  |  |
|  |  |  |  |  |  |  |  |

**Supplementary Figure S1**


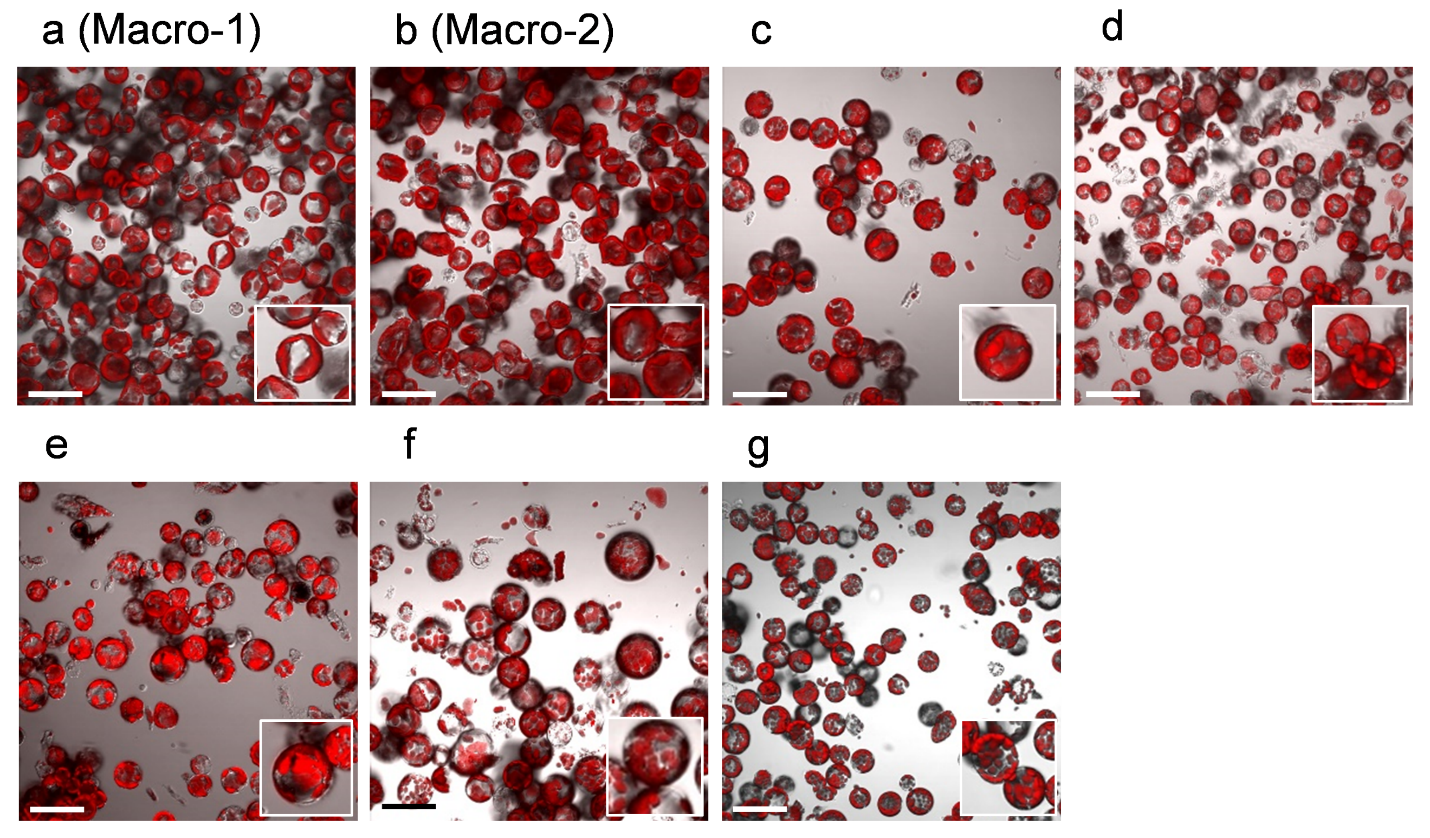


| **Line** | **Mean (µm)** | **SD** | **N. measured** |
| --- | --- | --- | --- |
| a (Macro-1) | 17.27 | 11.9 | 720 |
| b (Macro-2) | 16.81 | 9.61 | 786 |
| c | 11.17 | 5.43 | 1123 |
| d | 12.83 | 7.96 | 1649 |
| e | 14.82 | 9.06 | 843 |
| f | 14.38 | 8.66 | 938 |
| g (Wild-type) | 6.73 | 2.06 | 1382 |

**Figure S1: Confocal images showing chloroplast morphology in protoplasts from *AtFtsZ1* potato lines.** Confocal images (including insert images) showing chloroplasts into leaf protoplasts of six independent *AtFtsZ1* lines (a-f: Macro line 1-6, respectively), along with protoplasts from wild-type potato leaves (g). The six *AtFtsZ1* lines show enlarged chloroplast size (µm) comparing to wild-type potato plants (h). Results are expressed as mean ± SD (standard deviation) of the indicated number of measurements (N). Means were compared using ANOVA and when significant, mean separations were analyzed using Tukey HSD (p<0.05). Statistical significance is indicated by letters (a, b, c, d or e). Macro 1 and 2 (a and b, respectively) have been chosen for the following experiments. Confocal images are merge images of chlorophyll signal (red) and bright-field (gray). Scale bars: 50 µm; insert size: 50 µm.

**Supplementary Figure S2**


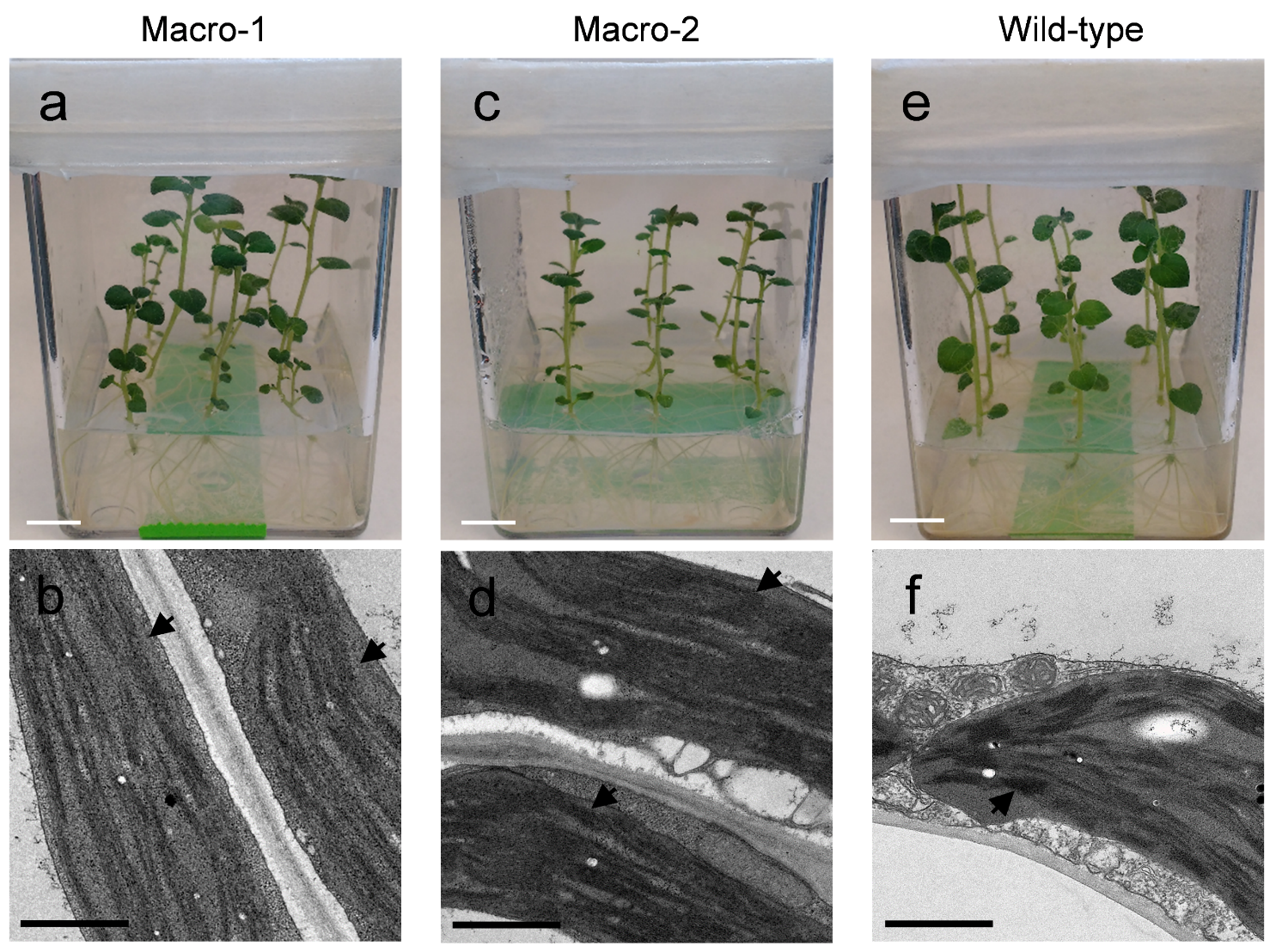


**Figure S2. Chloroplast morphology in *AtFtsZ1* lines and wild-type potato.** Three-week-old *AtFtsZ1* lines (Macro line 1 and 2) and wild-type control plants along with the ultra-structures of chloroplasts are shown (a-b, c-d and e-f, respectively). Thylakoids from macro-chloroplasts (b, d) and from wild-type controls (f) are indicated with black arrows. Electron micrographs of ultrathin sections showing mesophyll cells from leaf tissue prepared by chemical fixation. Scale bars: 10 mm (a, c, e); Scale bars = 1 µm (b, d, f).

**Supplementary Figure S3**


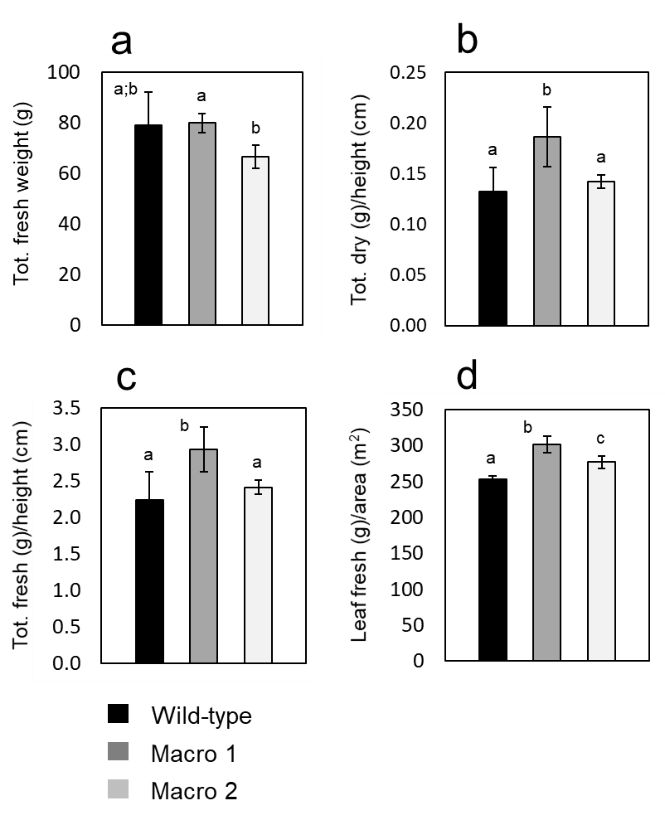


**Supplementary Figure S3: Growth characteristics of *AtFtsZ1* over-expressing potato lines at anthesis.** Histograms represent various plant characteristics at anthesis (time 1) of macro-chloroplast lines (Macro 1 and 2) and wild-type controls: (a) total fresh weight; (b) ratio of total dry weight to height; (c) ratio of total fresh weight to height; (d) ratio of fresh weight of leaves to their foliar area. The results as expressed as mean ± standard deviation of six plants per each genotype. Means were compared using ANOVA and when significant, mean separations were analyzed using Tukey HSD (p<0.05). Statistical significance is indicated by letters (a, b or c).

**Supplementary Figure S4**


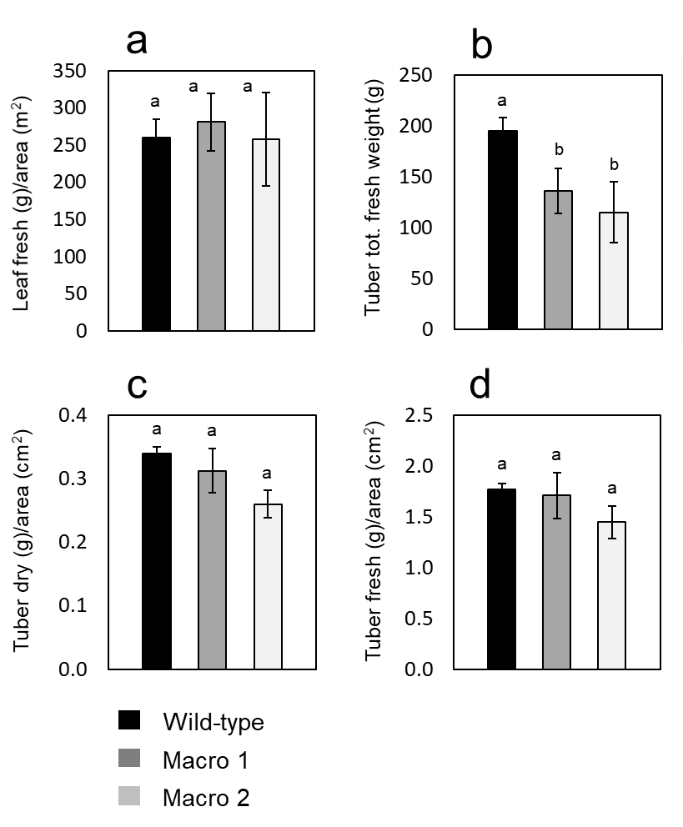


**Supplementary Figure S4: Growth characteristics and tuber phenotype of *AtFtsZ1* over-expressing potato lines at the end of life cycle.** Histograms represent various plant characteristics and tuber phenotype at the end of life cicle (time 2) of macro-chloroplast lines (Macro 1 and 2) and wild-type controls: (a) ratio of fresh weight of leaves to their foliar area; (b) total fresh weight of tubers; (c) ratio of total tuber dry weight to section area; (d) ratio of total tuber fresh weight to section area. The results as expressed as mean ± standard deviation of four plants per each genotype. Means were compared using ANOVA and when significant, mean separations were analyzed using Tukey HSD (p<0.05). Statistical significance is indicated by letters (a or b).

**Supplementary Figure S5**


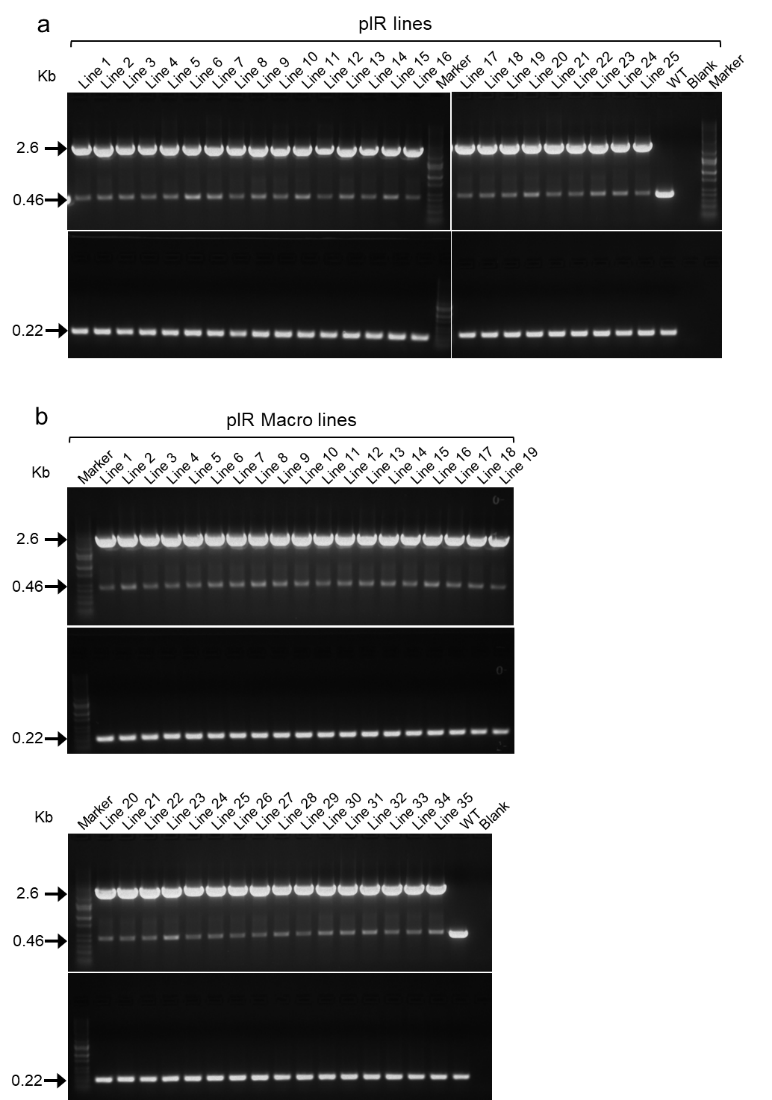


**Figure S5: PCR screening of the second round of pIR transplastomic lines.** Integration of the pIR vector in the plastome of normal and macro-chloroplasts lines, respectively (a and b). Pairs of primers specific for the *trnI/trnA* site were used to check integration in 25 normal pIR and 35 macro-chloroplasts pIR lines, respectively (a and b). DNA bands of 2.6 kb indicate correct integration, whereas 0.46 kb-bands indicate the presence of wild-type *trnI/trnA* site. PCR reactions specific for the *rbcL* gene (0.22 kb) were used as loading controls. Wild-type samples, blanks and molecular-weight markers are also shown in the gels.

**Supplementary Figure S6**


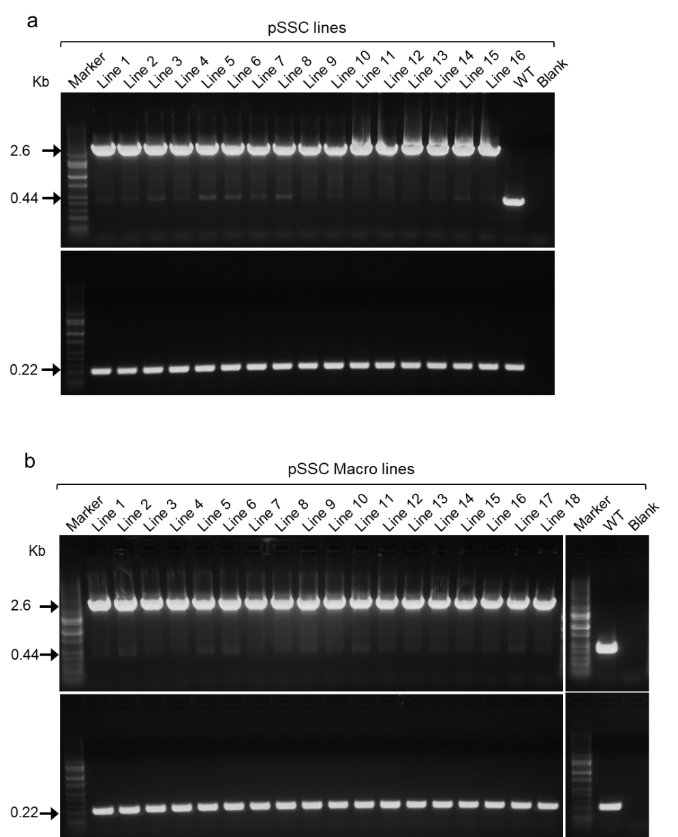


**Figure S6: PCR screening of the second round of pSSC transplastomic lines.** Integration of the pSSC vector in the plastome of normal and macro-chloroplasts lines, respectively (a and b). Pairs of primers specific for the *ndhG/ndhI* site were used to check integration in 16 normal pSSC and 18 macro-chloroplasts pSSC lines, respectively (a and b). DNA bands of 2.6 kb indicate correct integration, whereas 0.44 kb-bands indicate the presence of wild-type *ndhG/ndhI* site. PCR reactions specific for the *rbcL* gene (0.22 kb) were used as loading controls. Wild-type samples, blanks and molecular-weight markers are also shown in the gels.

**Supplementary Figure S7**


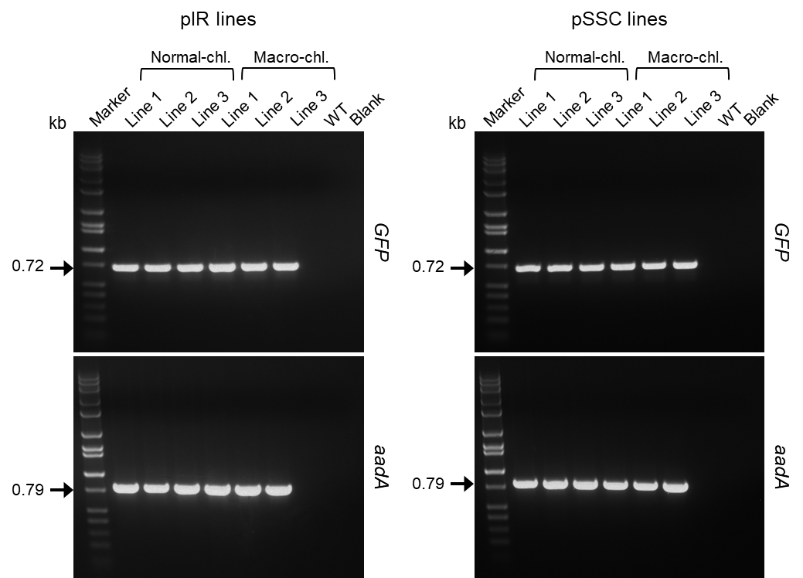


**Figure S7: Presence of transgenes in transplastomic lines.** Pairs of primers specific for either *GFP* or *aadA* were used to check the presence of full-length transgenes in pIR and pSSC transplastomic lines. The same three lines (1-3) for each construct (pIR or pSSC) and genotype (normal or macro) shown in Fig. 4 were tested. PCR bands of 0.72 and 0.79 kb confirm the presence of both *GFP* and *aadA* in all analyzed lines. Wild-type samples (WT), blanks and molecular-weight markers (kb) are also shown in the gels.

**Supplementary Video S1**

**Growth characteristics of transplastomic lines.** Video showing real time growth of pSSC and pIR transplastomic lines of both genotype, macro (M) and normal (N) size chloroplasts, along with wild-type control plants (WT). Two nuclear transgenic lines expressing GFP (Nuc-1 and 2) has also been used as comparison.
